# Immunology of amyotrophic lateral sclerosis – role of the innate and adaptive immunity

**DOI:** 10.3389/fnins.2023.1277399

**Published:** 2023-11-30

**Authors:** Stefan Mimic, Başak Aru, Cemil Pehlivanoğlu, Hadi Sleiman, Pavle R. Andjus, Gülderen Yanıkkaya Demirel

**Affiliations:** ^1^Centre for Laser Microscopy, Institute of Physiology and Biochemistry “Jean Giaja”, Faculty of Biology, University of Belgrade, Belgrade, Serbia; ^2^Immunology Department, Faculty of Medicine, Yeditepe University, Istanbul, Türkiye; ^3^Faculty of Medicine, Yeditepe University, Istanbul, Türkiye

**Keywords:** amyotrophic lateral sclerosis, neuroimmunology and neuropathy, innate immune system, adaptive immune system, neurodegeneration

## Abstract

This review aims to summarize the latest evidence about the role of innate and adaptive immunity in Amyotrophic Lateral Sclerosis (ALS). ALS is a devastating neurodegenerative disease affecting upper and lower motor neurons, which involves essential cells of the immune system that play a basic role in innate or adaptive immunity, that can be neurotoxic or neuroprotective for neurons. However, distinguishing between the sole neurotoxic or neuroprotective function of certain cells such as astrocytes can be challenging due to intricate nature of these cells, the complexity of the microenvironment and the contextual factors. In this review, in regard to innate immunity we focus on the involvement of monocytes/macrophages, microglia, the complement, NK cells, neutrophils, mast cells, and astrocytes, while regarding adaptive immunity, in addition to humoral immunity the most important features and roles of T and B cells are highlighted, specifically different subsets of CD4^+^ as well as CD8^+^ T cells. The role of autoantibodies and cytokines is also discussed in distinct sections of this review.

## 1 Introduction

First described by Jean-Marie Charcot, amyotrophic lateral sclerosis (ALS), also known as Lou Gehrig’s disease, is an irreversible neurodegenerative disease affecting both upper and lower motor neurons that progresses over time (Henkel et al., 2014). ALS is an adult-onset disease, most often occurring in men and women under the age of 60 years. The disease leads to very progressive and irreversible neurodegeneration of the upper and lower motor neurons, resulting in muscle weakness, dysarthria, and difficulty to swallow (dysphagia). Patients die within 4–6 years after the onset of the disease. The incidence of the disease is about two per 100,000 people (Logroscino et al., 2010). Although the etiopathogenesis of ALS remains unknown and inadequately studied, it is widely recognized as a complex and multifactorial condition, with immunological mechanisms playing an important role. Namely, there are two forms of ALS: the sporadic form (sALS), which is the most common with an unknown cause, and the familial form (fALS). In fALS, there is a disruption of the genes that code for axonal transport, vesicular traffic, or there occurs a disruption in RNA processing. The hereditary form of ALS is primarily associated with a specific mutation found in the gene responsible for producing superoxide dismutase type 1 (SOD1). It accounts for 20% of all known mutations, and transgenic mouse models of these human SOD1 mutations have provided an opportunity to investigate the disease mechanisms (Motataianu et al., 2020). SOD1 (Cu, Zn SOD) is a widespread cytosolic enzyme that converts the highly toxic superoxide anion into hydrogen peroxide. However, there are other mutations in focus of recent research, such as mutation in TAR-DNA-binding protein 43 (TDP43), FUS (Fused in Sarcoma), Angiogenin, and hexanucleotide repeats in the gene that codes for C9ORF72. TDP43 is encoded by the TARDP gene and cytoplasmic aggregation of the mutated forms of TDP43 protein are encountered in more 95% than all ALS cases (Neumann et al., 2006). Previously reported to be extensively expressed in peripheral myeloid cells and microglia, C9ORF72 mutations account for the cases of ~40% of fALS and 5–10% of non-fALS (DeJesus-Hernandez et al., 2011; Renton et al., 2011).

**Figure 1:**
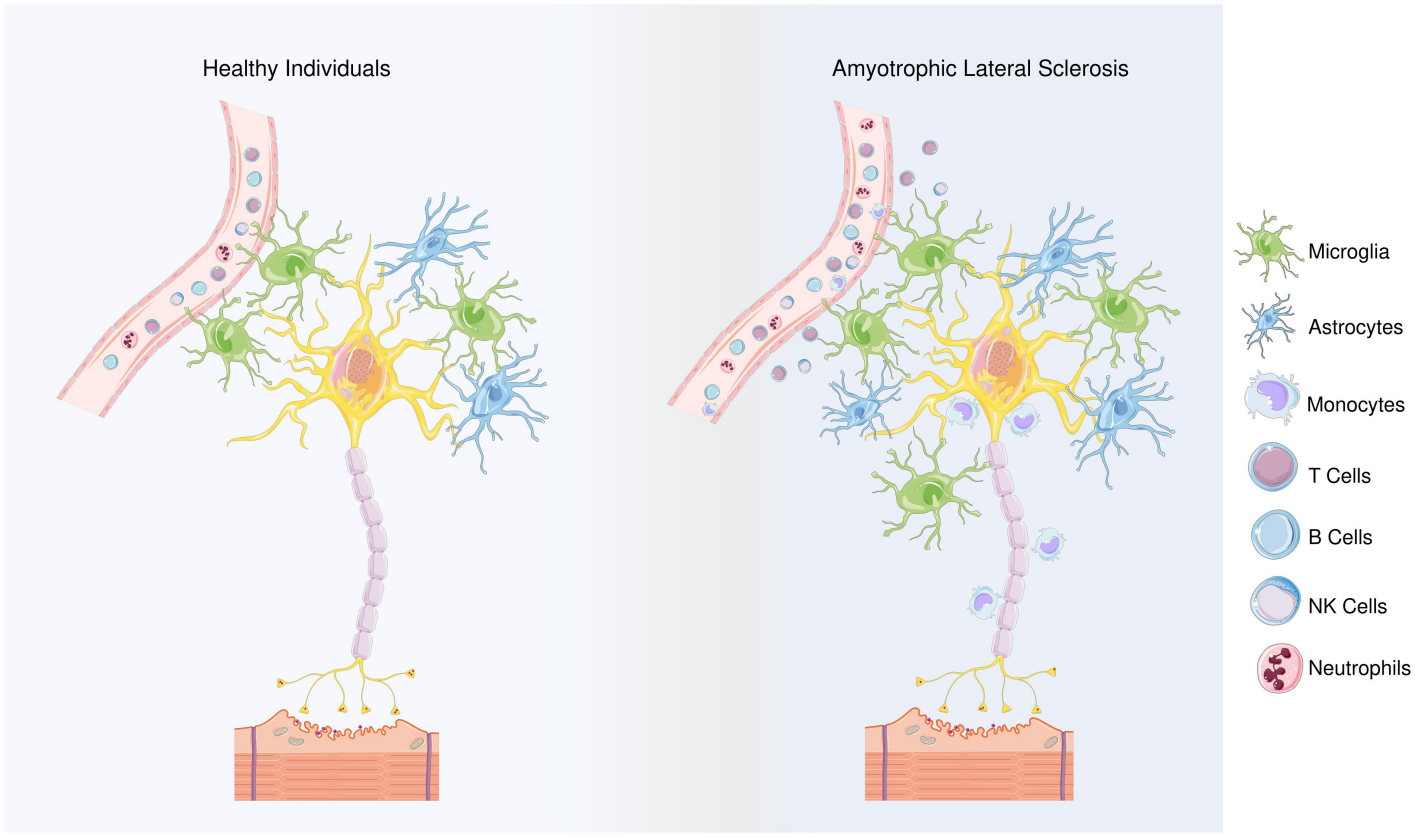
Differences between healthy individuals and ALS patients regarding the types of inflammatory cells. Members of the adaptive innate immune system, T and B cells play role in ALS progression, though their involvement may depend on the stage of the disease. Microglia and astrocytes are considered as the main contributors to the non-cell autonomous mechanism in ALS. The neuroprotective M2 microglia provide protection in the beginning of the disease by releasing anti-inflammatory mediators. However, as the disease progresses, a transition from the M2 to the neurotoxic M1 phenotype is observed. Microglial polarization in ALS is regulated with T cells and astrocytes’ responses. In terms of the first, anti-inflammatory cytokines IL-4 and IL-10 are released by Th2 cells and T_regs_, during the early stage of disease, in addition, Th2 cells also secrete various neurotrophic factors while as the disease progresses, Th1 cells release the pro-inflammatory mediators and contribute to the M1 polarization. Similarly, the NF-κB activation in spinal cord astrocytes can promote the neuroprotective phenotype of microglia and inhibit disease progression, but prolonged NF-κB activation in the later stages of the disease promotes pro-inflammatory microglial responses. In addition, impairments in glutamate transporters limits glutamate uptake of astrocytes, ultimately leading to dysfunction of motoneurons. Besides microglia and astrocytes, NK cells and monocytes can infiltrate the region and contribute to the inflammatory response. In the peripheral blood of the ALS patients, neutrophil percentages were reported to be increased, and the increase in their ratio to lymphocytes correlates with the disease progression. By releasing hematopoietic serine proteases, neutrophils also regulate NK cell toxicity.

Alterations in both innate and adaptive immune cell populations have been shown to influence disease progression in both mouse models and ALS patients (Henkel et al., 2006; Beers et al., 2008; Finkelstein et al., 2011; Butovsky et al., 2012; Lam et al., 2016; Zondler et al., 2016; Jin et al., 2020; Figure 1). In the case of ALS, Wallerian degeneration can occur due to the progressive degeneration of motor neurons and axons in the spinal cord (Beirowski et al., 2005; Coleman and Höke, 2020). This degeneration can lead to the loss of communication between the motor neurons and the muscles they control, ultimately resulting in muscle weakness, atrophy, and paralysis. The exact mechanisms of Wallerian degeneration in ALS are still not fully understood, but it is believed to be related to the accumulation of abnormal proteins within the motor neurons, such as TDP43 and FUS. These abnormal proteins can lead to the formation of aggregates and disrupt normal cellular processes, eventually resulting in the degeneration and death of the motor neurons. Furthermore, Wallerian degeneration can also cause the activation of neuroinflammatory processes, which further contribute to the progression of ALS. The immune system has been shown to play an important role in Wallerian degeneration as immune cells infiltrate the degenerating nerve, clear debris, and support axonal regeneration (Rotshenker, 2011). Herein, we aim to summarize the involvement of immune system in the pathogenesis of ALS.

## 2 The role of innate immunity in ALS

### 2.1 Monocytes and macrophages

Monocytes originate from the bone marrow, where they maturate upon colony stimulating factor 1 receptor stimulation (CSF-1R) from hematopoietic precursors common to monocytes, certain subsets of macrophages, and dendritic cells (Auffray et al., 2009). Several studies clearly indicate an increased infiltration rate of peripheral monocytes in mouse models of ALS and ALS patients’ spinal cords (Butovsky et al., 2012; Zondler et al., 2016) as well as their activation in peripheral blood (Mantovani et al., 2009). Activated monocytes from individuals with ALS show altered secretion of pro-inflammatory cytokines, altered adhesion behavior, and impaired phagocytosis (Zondler et al., 2016, 2017).

A growing number of scientific studies suggest that monocytes can infiltrate the brain and spinal cord during pathological conditions and their role is associated with the disruption of the blood–brain barrier (Cronk et al., 2018). When considering animal models, in mice, peripheral monocyte infiltration is associated with better motor neuron survival (Zondler et al., 2016). In another *in vivo* study, Ly6C^high^ monocytes have been shown to play an important role in disease progression and are associated with motor neuron injury (Butovsky et al., 2012).

Some of the most important pro-inflammatory mediators secreted by monocytes are chemokines C-X-C Motif Chemokine Ligand 1 (CXCL1) and C-X-C Motif Chemokine Ligand 2 (CXCL2), as well as FosB Proto-Oncogene, AP-1 Transcription Factor Subunit, Interleukin (IL)-1β, and Il-8, which unequivocally indicates that monocytes tend to polarize towards a pro-inflammatory phenotype in ALS (Du et al., 2020). Murdock and colleagues suggested that cell surface marker CD16 expression is altered during ALS, however, no correlation was reported between CD16^+^ monocytes and the ALS patient score, ALSFRS-R (Murdock et al., 2016). Figueroa-Romero and colleagues reported increased CCRL2 and CCR3 levels within the CNS (Figueroa-Romero et al., 2012). A comparison between the monocyte subsets indicated that CCRL2 expression is increased in CD16^+^ monocytes, however, no changes were reported in the expression pattern of CCRL2 in CD16 monocytes (Murdock et al., 2016). On the other hand, a significant difference was observed between CD16^−^ and CD16^+^ monocytes in terms of CCR3 expression: CD16^−^ monocytes have been reported to express significantly lower levels of CCR3. Moreover, a lack of correlation was reported between CCRL2 or CCR3 expression and the ALSFRS-R score. These data suggest that CCRL2 and CCR3 expression levels on CD16^−^ monocytes cannot predict the stage of the disease.

In summary, studies involving individuals with ALS revealed that peripheral monocytes are activated and polarized towards the M1 phenotype rather than the M2 phenotype. However, additional studies are still needed in order to clarify the role of peripheral monocytes and macrophages in the pathogenesis of ALS at the molecular-immunological level. Despite the absence of a clear correlation between the ALSFRS-R score and peripheral monocyte markers, it may be beneficial to explore additional markers expressed on monocytes to uncover potential connections between the molecular dynamics of monocyte subsets and ALSFRS-R scoring.

### 2.2 Microglia

Microglia are a dynamic population of glial cells that can have either neurotoxic or neuroprotective effects. Neuroinflammation is mainly observed at the sites of motor neuron injury, where activated and proliferating microglia, as well as dendritic cells (the most important antigen-presenting cells), are located (Henkel et al., 2009; Philips and Robberecht, 2011). *In vitro*, microglia can be activated by lipopolysaccharide (LPS), which binds to cell surface receptors CD14 and Toll-like receptor (TLR) 2/4, resulting in the release of reactive oxygen species (ROS) and mediating the excitotoxic effects of glutamate and calcium (Zhao et al., 2006).

**Figure 2:**
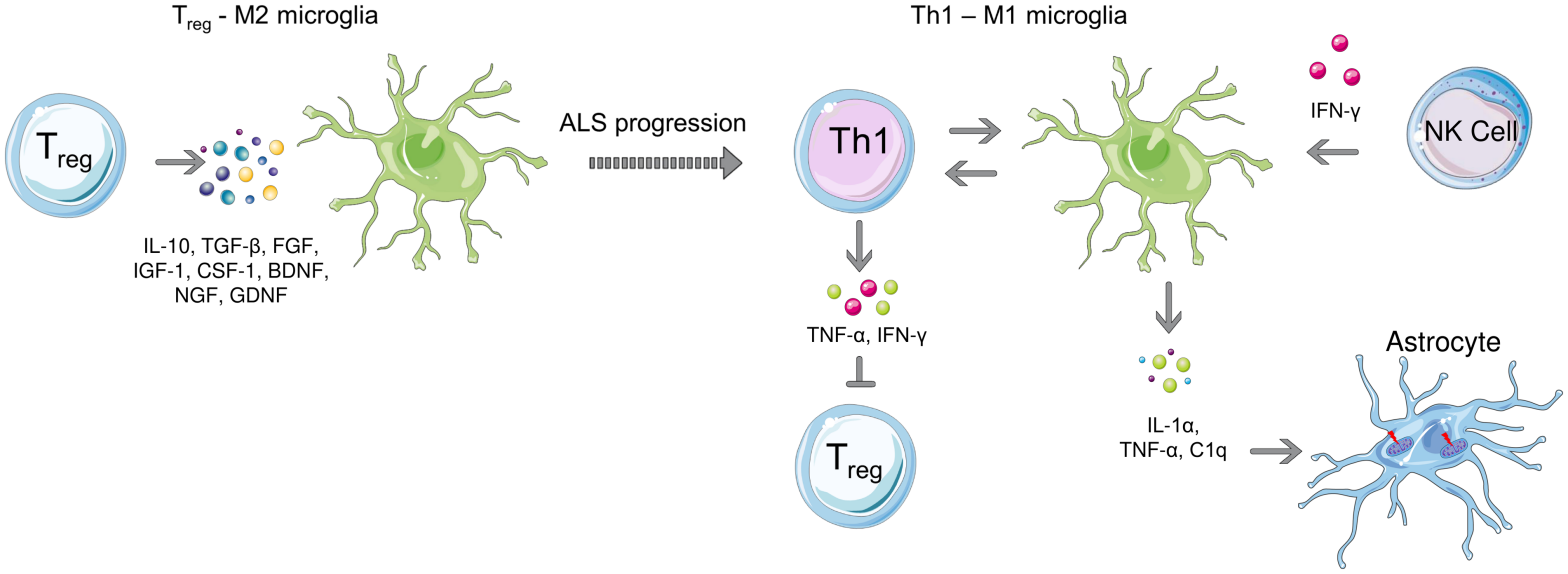
Microglial polarization during early **(left)** and late stages **(right)** of ALS. Anti-inflammatory microglia protect motor neurons at the beginning of the disease, while a transition to the neurotoxic M1 phenotype is observed as the disease progresses. T cell-microglia interaction plays role in microglial polarization, NK cells also contribute to the M1 polarization by secreting IFN-γ. Activated microglia release, IL-1α, TNF-α as and C1q which induce neurotoxic responses of astrocytes.

Microglial cells are resident macrophages of the CNS, and they participate in synaptic remodeling, pruning, and promotion of the formation of blood vessels (angiogenesis) (Du et al., 2017; Jurga et al., 2020). In the adult brain, microglia monitor signals from the environment and initiate inflammatory responses upon danger signals, providing the first line of defense in the brain. Microglia can recognize very subtle changes in the environment due to their surface receptors (Jurga et al., 2020). Although considered today as and oversimplified by analogy to peripheral monocytes, microglia subtypes are traditionally divided in M1 and M2 polarization phenotypes (Lyu et al., 2021). As observed in other diseases, it has been reported that microglia initially play a beneficial role before switching to a negative role in the advanced disease state (Henkel et al., 2009). Various studies have shown that M2 microglia protect motoneurons at the very beginning of ALS, while as the disease progresses, there is a transition from the M2 phenotype to the M1 phenotype (neurotoxic) (Figure 2; Liao et al., 2012; Gravel et al., 2016). This polarization arises from the crosstalk between T cells and microglia and will be discussed in more detail. In an *in vivo* study involving mSOD1 mice, the M2 phenotype of microglia with neuroprotective characteristics was more prevalent compared to their M1 counterparts in the early stages of the disease (Zhao et al., 2006). Activated microglia release inflammatory cytokines IL-1α and TNF-α as well as complement component 1q (C1q) which induce neurotoxic responses of astrocytes (Liddelow et al., 2017). Studies on primary microglia-motoneuron co-cultures have shown the neuroprotective characteristics of M2 microglia (Zhao et al., 2006) by secreting anti-inflammatory cytokines such as IL-10, transforming growth factor beta (TGF-β), fibroblast growth factor (FGF), IGF-1, CSF-1, brain-derived neurotrophic factor (BDNF), nerve growth factor (NGF), GDNF, and various neurotrophins (Colonna and Butovsky, 2017). However, as the disease progresses, polarization towards the neurotoxic M1 phenotype was observed, especially in the terminal stage of ALS (Henkel et al., 2014).

The essential role of microglia in the pathogenesis of motor neuron injury was also demonstrated in PU.1 knockout mice (PU.1−/−) which lack macrophages, neutrophils, T and B cells, and microglia (Beers et al., 2006). Upon bone marrow transplantation from mSOD1 mice, expression of mSOD1 within the CNS microglia was observed in PU.1−/− mice without clear signs of motor neuron injury or damage in ALS while bone marrow transplantation from healthy mice led to a reduction in motoneurons. In this study, the authors point out the extraordinary complexity of microglia by simultaneously expressing both neuroprotective and neurotoxic factors in ALS mice (Chiu et al., 2013).

Microglia exhibit characteristics of immature myeloid cells and undergo specific changes, such as morphological modifications and an activation of their functions, in response to signals from the environment or injury. Thus, they become mature myeloid cells that may present antigens and secrete cytokines and growth factors. Additionally, microglial cells can produce reactive oxygen species (Cardona et al., 2006). The CX3CR1 fractalkine receptor has been shown to play a role in microglial neurotoxicity in the SOD1G93A model of ALS. As the ligand CX3CL1 is expressed in neurons and the receptor CX3CR1 is expressed in microglia, it is possible to talk about signal transduction from neurons to microglia via the fractalkine receptor (Harrison et al., 1998). Future research should focus on the interactions of CX3CL1 and CX3CR1 to clarify the signal transduction mechanism between neurons and microglia, especially regarding the oxidative stress caused by microglial cells, considering that ALS is also associated with oxidative stress.

In addition to the traditional M1/M2 phenotype, in response to brain injury, a distinctive subset of microglia that expresses CD11c, the dendritic cell marker is observed (Sato-Hashimoto et al., 2019). The first study classifying CD11c + cells in the CNS as microglia dates back to 2006, where the authors have observed CD11c + cell populations in an Alzheimer’s Disease (AD) mouse model identified as microglia (Butovsky et al., 2006). Interestingly, in a study performed on a mice ALS model, authors have discovered a distinct signature for these cells in the final stages of the disease in comparison to microglia from healthy brains where the microglia were reported to have CD11c expression (Chiu et al., 2013). In another study, authors have reported the presence of so-called “disease-associated microglia” (DAM) in an *in vivo* AD model which is characterized by the reduced expression of homeostatic genes (Keren-Shaul et al., 2017). DAM have been found in the surrounding of the Aβ plaques, indicating these cells take place in scavenging of extracellular aggregates and cellular debris. DAM were not only observed in AD but also in the spinal cord of the ALS model SOD1G93A mice. In ALS patients, a similar gene expression pattern was detected in the motor cortex, suggesting that DAM may be a general response to the protein accumulation in neurodegeneration. It is still unclear, though, whether these cells are a component of a neuroprotective glial response that is activated late during ALS to mitigate the disease, or if it is instead an excessively reactive response to neuronal death that ultimately contributes to amplifying the damage in the affected CNS regions (Cipollina et al., 2020). Recently, Takahashi revealed the presence of two microglia subgroups of spinal cord lesions in ALS that can be pathologically distinguished according to their TMEM119 expression (Takahashi, 2023). The TMEM119+ microglia group also exhibited expression of the microglial activation marker CD68 and endothelial activation, indicating the presence of inflammatory processes in ALS lesions, and the author have suggested that these cells may represent DAM-independent inflammatory neurodegeneration as DAM suppresses the expression of TMEM119.

### 2.3 Complement system

The complement system is a complex series of plasma proteins that work together to generate fragments of C3 and C5 proteins through a sequence of cleavage. Recent studies have identified the involvement of the classical pathway of the complement system in the development of amyotrophic lateral sclerosis (ALS), with increased levels of C1q and C4 components observed in the cerebrospinal fluid, CNS, serum, and skeletal muscles of ALS patients (Apostolski et al., 1991; Tsuboi and Yamada, 1994; Yamada et al., 1994; Sta et al., 2011; Bahia El Idrissi et al., 2016). Animal models of ALS have also confirmed the involvement of the upstream pathways of the complement system (Perrin et al., 2005; Ferraiuolo et al., 2007; Lobsiger et al., 2007). However, the role of the terminal component C5 must also be considered, as increased levels of factor C5a and terminal complement complex (C5b-9) have been reported in ALS patients (Mantovani et al., 2014).

Upregulation of C5aR1 signaling has been identified as a potential contributor to motor neuron death in ALS patients, with increased immunostaining for C5aR1 in motor neurons of ALS patients (Bahia El Idrissi et al., 2016). Furthermore, biopsy samples from ALS patients have detected the presence of C59a, a major regulator of the membrane attack complex (Bahia El Idrissi et al., 2016). Despite these findings, further studies are required to fully understand the complex involvement of the complement system in the pathogenesis of ALS, particularly regarding the lack of human studies on the upstream part of the complement system and the C3 component.

### 2.4 Natural killer cells

Natural killer cells (NK cells) represent the most abundant subset of innate lymphoid cells, and they take part in anti-tumor and anti-viral defense. NK cell function is regulated by a balance between signals from activating and inhibitory receptors. Unlike cytotoxic CD8^+^ T cells, NK cells do not require prior antigen exposure for an anti-tumor response (Abel et al., 2018). NK cells participate in both innate and adaptive immunity. Thus, NK cells infiltrating the CNS regulate neuroinflammatory processes in neurodegenerative diseases (Infante-Duarte et al., 2005; Poli et al., 2013; Hertwig et al., 2016). NK cells are very important given the fact that motoneurons are sensitive to NK cells during ALS (Song et al., 2016).

NK cells are reported during end-stage disease in the spinal cord of mSOD1 mice (Beers et al., 2008). In addition, an increased number of NK cells was observed in the blood of ALS patients (Beers et al., 2017). Motoneuron degeneration in ALS is associated with dysregulated neuroinflammatory microcirculation (Barbeito et al., 2010; Thonhoff et al., 2018a,b) or microglial activation, which impairs axonal regeneration (Nardo et al., 2016; Spiller et al., 2018). When considering these findings, the degeneration of motor neurons may stem from direct cell-to-cell contacts with NK cells, which release perforin and granzyme B, exerting a direct neurotoxic effect on motor neurons, as well as highlighting the role of innate immunity in neurodegeneration associated with ALS (Garofalo et al., 2020).

The presence of NK cells in the motor cortex and spinal cord of post-mortem samples of ALS patients, as well as the presence of NKG2D ligands expressed on motoneurons, has been previously reported (Garofalo et al., 2020). In this study, it has been demonstrated that NK cells are neurotoxic to motor neurons that express NKG2D ligands in an animal model of ALS. In the mouse model of fALS, it has been shown that NK cells localize in the motor cortex and spinal cord, where the reduction in the number of NK cells leads to attenuation of motoneuron degeneration; the latter was also confirmed on hSOD1G93A and TDP43A315T rodent models (Garofalo et al., 2020). NK cells also secrete the pro-inflammatory IFN-γ, which promotes polarization towards the M1 phenotype of microglia. Increased levels of IFN-γ were detected in hSOD1G93A mice compared to WT (Garofalo et al., 2020). In this study, patients with sALS were investigated in terms of the presence of NK cells as well as the frequency of their subtypes. NKp46^+^ cells were detected in the motor cortex and spinal cord of ALS individuals, in contrast to control subjects. A reduction of circulating CD56^+^/CD3^−^ cells was also observed in the peripheral blood of sALS patients. The fractalkine receptor CX3CR1 plays an important role in the migration of NK cells in the CNS (Garofalo et al., 2020) as a member of the NKp protein family (Moretta et al., 2000); CXCR3^+^ expression was shown to be correlated with ALS progression (Murdock et al., 2021a).

Interestingly, NK cells contribute to ALS progression in a sex and age dependent manner. In SOD1G93A mice, NK depletion extended survival in female but not male mice (Murdock et al., 2021a). Moreover, male mice had higher levels of microglia in the CNS which was decreased upon NK depletion. In ALS subjects, a correlation between NKG2D or NKp46 and ALSFRS-R was reported in older patients while a correlation between NKp30 and ALSFRS-R was observed in women, suggesting age and gender specific activation patters of NK cells. Although these data suggest immune responses may be based on sex and age affect the progression of ALS, the underlying mechanisms causing these variations remain unknown. Nevertheless, numerous reports also imply that hormones can directly affect immune responses, including NK cells, and since plasma levels of testosterone and estrogen differ between men and women and change with age or the onset of menopause, sex hormones are the most likely cause of the observed alterations (Murdock et al., 2021a).

### 2.5 NKT cells

NKT cells are a subset of T cells that can recognize and respond to glycolipid antigens presented by CD1d molecules (Kawano et al., 1997). These cells are primarily found in the liver and are known for their role in regulating immune responses. In neuroinflammation, NKT cells are believed to play a crucial role due to the high lipid content in the CNS (Halder et al., 2007). Research has shown that the numbers of NKT cells are altered in several autoimmune diseases (van der Vliet et al., 2001; Hammond and Kronenberg, 2003; Grajewski et al., 2008). However, the exact role of NKT cells in other neurodegenerative diseases is still not well understood. Rentzos and colleagues have revealed that NKT levels are increased in peripheral blood of ALS patients (Rentzos et al., 2012). In an *in vivo* ALS model, mSOD1 mice, it was shown that the number of NKT cells was increased in the CNS as well as in lymphoid organs (Finkelstein et al., 2011). In this study, administration of α-galactosyl ceramide analogue PBS57 prolonged the survival of mSOD1 mice and alleviated the disease. The lipid antigen PBS57 also decreased the number of NKT cells in addition to hampering their responses. These findings indicate that targeting NKT cells may be a novel aspect in ALS treatment (Finkelstein et al., 2011). However, it should be noted that the therapeutic effect of NKT cell inhibition in ALS is not confirmed with clinical studies yet. Further research is needed to determine the potential of NKT cell-targeted therapies in the treatment of neurodegenerative diseases.

### 2.6 Neutrophils

Neutrophils are the most abundant population of leukocytes and play a role in inflammation as well as various autoimmune and inflammatory diseases (Németh et al., 2020). The role of neutrophils in ALS pathogenesis is reported to be controversial: some studies suggest that neutrophils have predominantly pro-inflammatory roles in disease pathogenesis and lead to disruption of the blood brain/spinal cord barrier (Weiss, 1989; Garbuzova-Davis and Sanberg, 2014), while other studies suggest that these cells play a role in neuroprotection and in repairing damaged neurons (Butterfield et al., 2006; Kim and Moalem-Taylor, 2011; Kurimoto et al., 2013). However, there are certain studies addressing that neutrophil to monocyte or lymphocyte ratios may serve as potential biomarkers in ALS.

Various studies indicate an increase in neutrophil percentages in ALS (Desport et al., 2001; Banerjee et al., 2008; Keizman et al., 2009; Chiò et al., 2014; Murdock et al., 2016). As the disease progresses, the ratio of neutrophils to monocytes was found to increase significantly, suggesting that this ratio might be considered as a predictor of the disease course (Murdock et al., 2016). In a more recent study, Leone et al. (2022) investigated the relationship between neutrophil-to-lymphocyte ratio (NLR) and ALS progression rate as well as survival and concluded that NLR may serve as a low cost, fast, and easy biomarker for assessing the disease course. It was suggested that prospective studies are required to elucidate the changes in NLR during the disease progression prior to proposing it as a biomarker in monitoring ALS. Noteworthy, in this study, the patients were not classified according to the genetic background of the disease. Wei et al. (2022) investigated the correlation between NLR and ALS progression in sALS patients by assigning the patients into three groups according to their NLR values and revealed that patients with high NLR had lower ALSFRS-R scores, faster disease progression rates, and older disease onset age. In line with the study published by Leone et al. (2022), these results also confirm that NLR may aid in predicting the disease progression and survival in sALS patients.

In terms of surface proteins expressed on neutrophils, Murdock et al. (2016) reported that the percentage as well as the number of CD16^−^ monocytes along with CCR3 and CCRL2 were decreased in ALS patients, however these findings did not correlate with the ALSFRS-R score. Another cell surface receptor that plays role in neutrophil chemotaxis and neuroinflammatory responses is CXCR2, which was previously found to be overexpressed in a specific subset of ALS patients as well as SOD1G93A mice at symptomatic stages (Aronica et al., 2015; Morello et al., 2017). Neutrophils may also affect other immune cell populations’ responses as well: cathepsin G and elastase released by these cells can promote NK cell cytotoxicity (Costantini and Cassatella, 2011). Similar to NK cells, neutrophils also have a sex-specific effect on disease survival, and the crosstalk between these two components of innate immune system can affect disease progression (Murdock et al., 2021b).

### 2.7 Mast cells

Being a cellular component of the innate immune system, mast cells are differentiated from hematopoietic myeloid precursors, and are found in all vascularized tissues including brain (Kovacs et al., 2021). Precursors of these cells are recruited to the tissues through a trans-endothelial passage and maturate in the local tissue microenvironment. Mast cells are one of the first cells to be activated in response to tissue damage, releasing mediators and enzymes (Abonia et al., 2005; Wernersson and Pejler, 2014). Mast cells can release cytokines, chemokines, leukotrienes, proteases, as well as bioactive polyamines, and play many important roles in pathogen clearance, allergic reactions, and intestinal cancer (Shea-Donohue et al., 2010).

Trias and coworkers revealed significant infiltration of neutrophils and degranulating mast cells in the skeletal muscles of ALS patients (Trias et al., 2018). These cells can interact with each other, muscle fibers, and motor plates, implying coordinated neuroinflammation that is associated with neuromuscular synapse denervation and muscular atrophy. Also, these cells are highly localized around neuromuscular junctions and along degenerating axons of ventral roots and sciatic nerves (Trias et al., 2018). In ALS, mast cells can cross the blood-spinal cord barrier and release various mediators including neuropeptides, proteases, cytokines, and histamine upon degranulation, leading to local neuroinflammation and dysregulated neuronal function (Jones et al., 2019).

In human ALS subjects, the number of mast cells are reported to increase in the quadriceps muscles. Furthermore, the positive correlation between the number of mast cells and neutrophils suggests that these cells act in a very complex and coordinated manner. Mast cells are recruited in response to motor neuron injury, while on the other hand, they can trigger the recruitment of neutrophils (Chen et al., 2001; Wezel et al., 2015). In patients, the density and size of mast cells are reported to be significantly greater compared to controls. Moreover, in ALS, mast cells are reported to localize near muscle fibers while in healthy controls, they are observed around blood vessels (perivascular localization).

Degranulation of mast cells in the muscles of ALS patients indicates multiple responses at the local level mediated by the release of cytokines, proteases, trophic factors, and vasoactive mediators (Krystel-Whittemore et al., 2015). In healthy individuals, mast cells take part in muscle repair. They also initiate damage to muscle fibers and motor plates, causing fibrosis and collagen deposition either directly or indirectly (Levi-Schaffer et al., 2002; Lee and Kalluri, 2010). Mast cells and neutrophils also invade the area of the endoneurium of the sciatic nerve up to the ventral roots (Trias et al., 2017). Chymase released by mast cells is a well-known chemoattractant cytokine for neutrophils that underlies the association between these two cell types (He and Walls, 1998; Tani et al., 2000). In addition to the mentioned factors, mast cells can release very different vasoactive and inflammatory mediators that exert harmful effects during the disease (Zhuang et al., 1996; Sprague and Khalil, 2009; Theoharides et al., 2012). Recently, it is reported that in both ALS patients as well as the murine models of the disease, clusters of mast cells expressing tyrosine kinase receptor c-Kit+ and other characteristic markers which lack the toluidine blue metachromasia are observed between motor neuron somas and nearby microvascular elements in the spinal cord (Jones et al., 2019). Moreover, expression of stem cell factor (SCF) is found to be overexpressed in the reactive astrocytes in ALS, which may act as a chemoattractant and lead to differentiation of mast cells. These findings suggest complex interactions between neurons, mast cells, and reactive microglia in disease pathology in addition to highlighting the potential usage of tyrosine kinase inhibitors in ALS.

The pathological significance of the recruitment of mast cells into the neuromuscular compartment remains unclear in ALS, although certain studies underline that chronic neuroinflammation could be a very important harmful factor (Chiu et al., 2009; Keizman et al., 2009; Martínez-Muriana et al., 2016). Overall, these studies reveal that mast cells and neutrophils are abundant along the peripheral motor pathway in ALS. Mast cells have harmful, cytotoxic effects for motor neurons, where they can be a pharmacological target of tyrosine kinase inhibitors, given that they express the c-Kit as mentioned above (Galli et al., 1993; Wernersson and Pejler, 2014; Jones et al., 2019). Pharmacological inhibition of mast cells with the drug masitinib reduces the level of motor deficit in rats, therefore implying the role of mast cells during disease progression and may be considered as an adjuvant therapy in ALS patients (Trias et al., 2017).

### 2.8 Astrocytes

Astrocytes are the most abundant type of glial cell in the central nervous system and are involved in many important functions, such as maintaining the blood–brain barrier, regulating neurotransmitter levels, and supporting neuronal function (Sofroniew and Vinters, 2010; Haidet-Phillips et al., 2011). In ALS, astrocytes were reported to undergo significant changes that contribute to the disease progression by producing excessive amounts of pro-inflammatory cytokines, leading to inflammation and damage to motor neurons (Philips and Robberecht, 2011). In addition to microglia, astrocytes are considered as one of the main contributors to the cell-autonomous mechanism in ALS (Stoklund Dittlau and Van Den Bosch, 2023).

**Figure 3:**
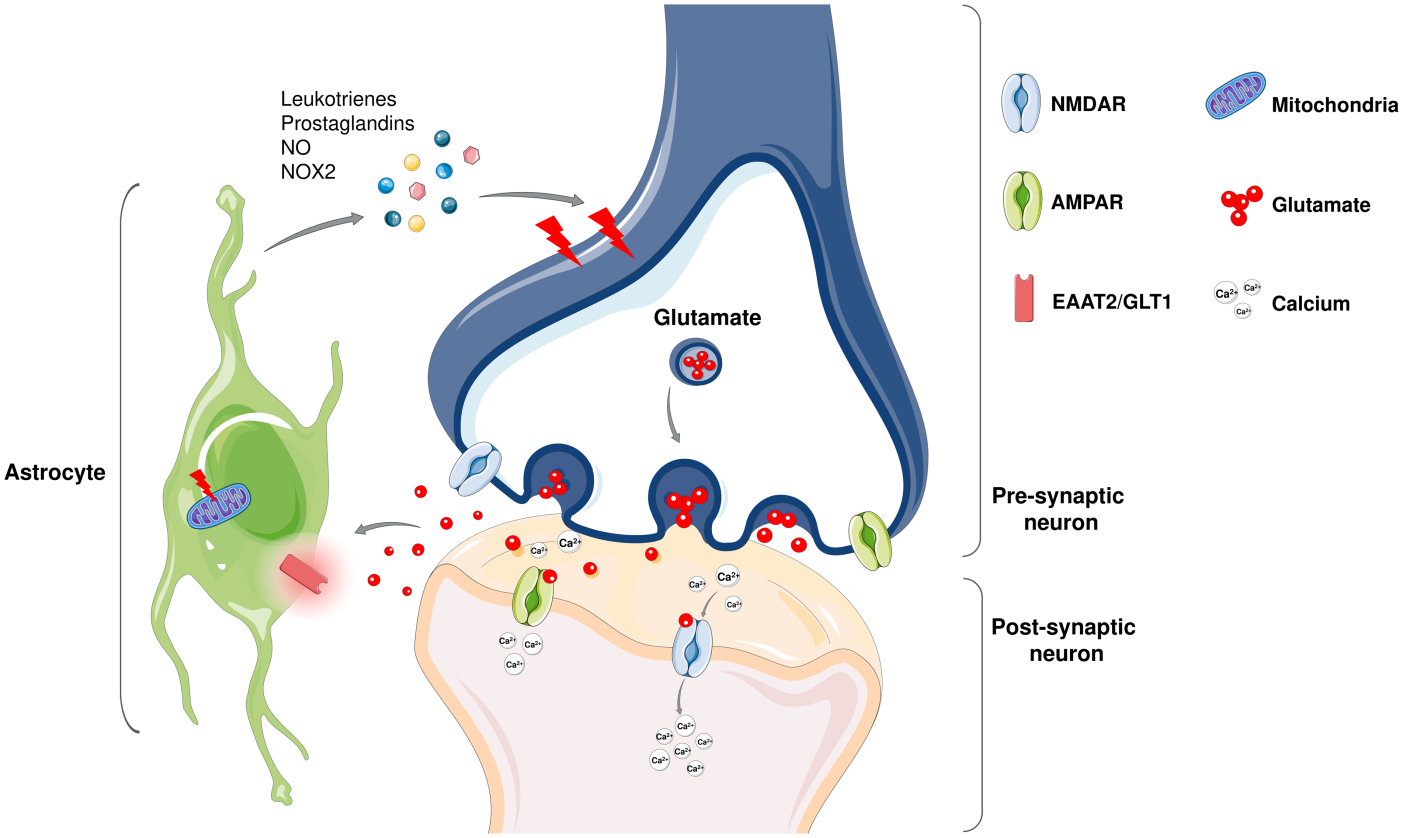
Even though ALS is considered as motor neuron disease, not only neurons but glial cells including astrocytes also play a role in the non-cell autonomous disease pathology (Stoklund Dittlau and Van Den Bosch, 2023). Under physiological conditions, astrocytes are involved in the modulation of neuronal synapse plasticity; ion and metabolic homeostasis; cellular communication; supporting blood–brain barrier and providing structural support. In healthy tissues, non-activated astrocytes remain immobile and support neuronal homeostasis while upon damage, they activate and initiate an immune response to fight against the harmful agents together with microglia. In neurodegenerative diseases including ALS, an abnormal astrocyte reactivity that contributes to neuronal death is observed. Being the most abundant excitatory neurotransmitter in the nervous system, glutamate is found at high concentrations in pre-synaptic nerve terminals where it binds to receptors such as α-amino-3-hydroxy-5-methy-4-isoxazolepropionic acid (AMPA) and *N*-methyl-D-aspartate (NMDA) receptors. Since high levels of glutamate may lead to a lethal increase in intracellular calcium astrocytes play a protective role in glutamate clearance from the synaptic cleft via excitatory amino acid transporters (EAATs). Among five EAATs identified, EAAT-1 and EAAT-2 are primarily expressed on astrocytes (Ng and Ng, 2022). Dysregulation of the human counterpart of GLT1, EAAT-2 (Rimmele and Rosenberg, 2016) expression has been shown to be altered in ALS and reported to contribute increased glutamate levels in the cerebrospinal fluid of ALS patients. Moreover, astrocytes produce inflammatory mediators that contribute to neuronal death (Stoklund Dittlau and Van Den Bosch, 2023). Mitochondrial dysfunction in astrocytes also promote neuronal death in ALS (Zhao et al., 2022).

Under normal conditions, astrocytes remove excess glutamate from the synaptic cleft via glutamate transporters. However, in *in vivo* ALS models as well as in ALS patients, the dysfunction of the glutamate transporter-2 (EAAT-2) leads to decreased uptake of glutamate from astrocytes, which in turn affects the function of motoneurons (Howland et al., 2002; Dunlop et al., 2003; Pardo et al., 2006; Papadeas et al., 2011). It has been shown that ALS astrocytes harbor mitochondrial dysfunction (Stoklund Dittlau and Van Den Bosch, 2023), can release inflammatory molecules and mediators such as leukotrienes, prostaglandins, nitric oxide (NO), and NAD(P)H-dependent oxidase NOX2 (Hensley et al., 2006; Marchetto et al., 2008; Haidet-Phillips et al., 2011), and can induce neuronal death by necroptosis (Re et al., 2014; Figure 3). Recent studies also reveal that exosomes released from mSOD1 astrocytes contain mutant SOD1 and have dysregulated miRNA profile that contributes to ALS pathology (Basso et al., 2013; Barbosa et al., 2021). Upregulated miR-155 was reported in ALS mice models in addition to fALS and sALS patients (Cunha et al., 2018). On the contrary, the decreased miR-494-3p release of C9ORF72 astrocytes led to neuronal network damage (Varcianna et al., 2019). Dying neurons also release certain miRNAs including miR-218 that downregulates glutamate transporter-1 that supports reactive astrocytes (Hoye et al., 2018).

Astrocytes have also been shown to support the survival and function of motoneurons as they release neurotrophic factors such as BDNF and ciliary neurotrophic factor (CNTF); in addition, these cells can provide energy substrates which are critical for normal neuronal functions (Moore et al., 2011; Allen and Lyons, 2018). However, Vargas and Johnson (2010) reported that while neurotrophic factors in mice slow the progression of ALS, they fail to provide neuroprotection in humans.

Besides their interactions with neurons, astrocytes can also modulate microglial responses as in the early stages of ALS as in the early stages of the disease, Nuclear factor kappa B (NF-κB) activation in astrocytes reside in the spinal cord can promote anti-inflammatory microglial activity while in later stages of the disease, prolonged activation of this pathway aggravates immune responses that results in pro-inflammatory microglial responses (Ouali Alami et al., 2018). According to our current knowledge, it is clear that astrocytes play important roles in ALS, although the sequence of events that lead to astrocyte dysfunction is still not fully elucidated (Liu and Wang, 2017).

In conclusion, astrocytes play a critical role in the pathogenesis of ALS. Dysfunctional astrocytes can contribute to inflammation, glutamate toxicity, and the formation of protein aggregates, all of which are key features of the disease. Further research on the role of astrocytes in ALS may lead to the development of new therapeutic approaches for this devastating disorder (Peric et al., 2017).

### 2.9 Microbiome

The microbiome is a collection of microorganisms that live within and on the human body and play an important role in human health. Recent studies suggest that alterations in the gut microbiome may be involved in the pathogenesis of several neurological diseases. A growing body of evidence suggests that dysbiosis, or an imbalance of the gut microbiome, may contribute to the development and progression of ALS.

Recently, Blacher and coworkers reported that ALS-prone Sod1 transgenic mice had alterations in their gut microbiome compared to healthy mice (Blacher et al., 2019). Wu et al. (2015) reported disrupted intestinal intercellular junction in SOD1G93A mouse model in addition to a decrease in butyrate-producing bacteria prior to the symptomatic stage. Zeng et al. (2020) reported alteration in the composition of the gut microbiota in addition to the metabolic products in ALS patients. However, in another study by Brenner and coworkers, it was found that ALS patients do not have a significantly different microbiome in terms of diversity and quantity compared to healthy controls, indicating the requirement of further research in order to reveal the connection between microbiome and ALS (Brenner et al., 2018).

The mechanisms by which the gut microbiome may contribute to ALS pathogenesis are not fully understood, but there is evidence that alterations in the gut microbiome may lead to increased gut permeability and the release of microbial toxins, which can trigger immune responses and inflammation that could contribute to the degeneration of motor neurons (Martin et al., 2022). In summary, there is emerging evidence suggesting that alterations in the gut microbiome may play a role in the pathogenesis of ALS. Further research is needed to fully understand the mechanisms underlying this relationship and to explore the potential for microbiome-targeted therapies for ALS.

## 3 The role of adaptive immunity in ALS

### 3.1 CD4^+^ T cells

CD4 T cells, also known as helper T cells, play a crucial role in orchestrating and regulating the immune response in ALS. These cells show several subsets with distinct functions: Th1, Th2, Th17 cells, and regulatory T cells. Th1 and Th17 subsets show evident pro-inflammatory characteristics releasing IL-6, IL-17, IL-21, IL-22, IL-23, IFN-γ, and TNF-α, while Th2 and regulatory T cells (T_regs_) show anti-inflammatory characteristics releasing IL-4, IL-10, and TGF-β (Motataianu et al., 2020).

In comparison with the healthy controls, ALS patients were reported to have increased levels of activated CD4^+^ T cells in CSF, which have an essential role in the early stage of the disease (Henkel et al., 2006; Beers et al., 2008; Chiu et al., 2008) (Rolfes et al., 2021). However, as the disease progresses, Th1 cells begin to exert their harmful effects, and elimination of these cells has been suggested as an effective approach for extending survival (Shao et al., 2013). In addition, the increase in Th1 cells is accompanied by a decrease in Th2 cells (Jin et al., 2020).

CD4^+^ T cells were observed at the sites of motoneuron injuries in mSOD1 transgenic mouse models and reported to promote neuroprotection and slow down the course of the disease (Motataianu et al., 2020). Role of T cells were also investigated by crossing mSOD1 mice with RAG2−/− mice, which are characterized by a lack of T and B cells, and it was observed that CD4+ T cells contribute to prolonging survival (Beers et al., 2008). However, the situation is more complicated, considering that CD4^+^ T cells with neurotoxic (Th1, Th17) and neuroprotective (Th2, T_reg_) phenotypes (Tiemessen et al., 2007; Beers et al., 2011) enter direct interaction and communication with microglia cells and macrophages. Accordingly, during the early stage of the disease, the anti-inflammatory cytokines released by Th2 cells in addition to T_regs_, IL-4 and IL-10 were increased (Tiemessen et al., 2007; Beers et al., 2011). Th2 cells also release neurotrophic factors such as TGF-β, BDNF, and GDNF, which are inversely correlated with the course of the disease (Motataianu et al., 2020). During this phase, regulatory T cells contribute to neuroprotection by interacting with microglia; however, as the disease progresses, the neuroprotective T_regs_-M2 phenotype changes to the Th1-M1 neurotoxic one, whereby Th1 cells release the pro-inflammatory cytokine IFN-γ (Chiu et al., 2008). Microglia and Th1 cells can secrete TNF-α, inhibiting regulatory T cells’ function through their transcription factor FOXP3 (Nie et al., 2013).

Previously, it has been shown that peripheral T cell counts are increased in a time-dependent manner in ALS (Murdock et al., 2017) and pro-inflammatory T cell populations are more prominent (Jin et al., 2020). The decrease in the CD4^+^ T cell levels, on the other hand, is in correlation with ALS progression and it was suggested that the reduced T cell amount may represent the decreased neuroprotection during ALS progression (Murdock et al., 2017). Previously, Rofles et al. reported elevated levels of CD4^+^ T cells in CSF (Rolfes et al., 2021). On the other hand, decreased relative numbers CD3^+^ caused by the net decreased by CD4^+^ cell number was reported in the peripheral blood of ALS patients compared to healthy controls (Ramachandran et al., 2023). Another study reported that while Th2-differentiated CD4^+^ central memory T cells were negatively associated with the risk of death in ALS patients, CD4^+^ effector memory re-expressing CD45RA T cells were positively correlated with risk of death (Cui et al., 2022).

Th17 cells play an important role in autoimmune diseases, and the role of these cells in ALS is becoming increasingly recognized. Investigating immune profiles of ALS patients, Jin and coworkers reported that T cell subtypes in peripheral blood shift towards Th17 in addition to Th1 phenotypes (Jin et al., 2020). Moreover, a moderate negative correlation between Th17 cells and ALSFRS-R was detected. The levels of IL-17 and IL-23 in CSF and serum have been found to be significantly increased in ALS patients compared to patients with non-inflammatory neurodegenerative diseases (Rentzos et al., 2010). Although the levels of these cytokines did not correlate with the progression of the disease, they are considered markers of inflammation and may be involved in the pathogenesis of ALS in humans. The increase in the cytokine IL-17 may reflect the activation of Th17 cells, suggesting that these cells may play a significant role in ALS pathogenesis. Moreover, in a recent study, it is revealed that motor neurons express IL17 receptors, and IL-17 can directly exert cytotoxicity on these cells which can be rescued by IL17 neutralization or anti-IL17RA treatment (Jin et al., 2021). It should be noted that measuring cytokine levels in the serum may have limitations, and it may not reflect the pathological processes occurring in the brain due to the short half-life of cytokines and their binding to the molecules in the bloodstream. Therefore, further research is needed to fully understand the role of Th17 cells and cytokines in the pathogenesis of ALS.

### 3.2 CD8^+^ T cells

CD8^+^ T cells, also known as cytotoxic or cytolytic T cells, are a crucial component of the adaptive immune system, and their role in the pathogenesis of ALS is an area of active research. In ALS, CD8^+^ T cells are thought to be involved in the direct killing of motor neurons through the MHC class I complex, as well as by secreting granzymes and perforins. Studies have found that the number of CD8^+^ T cells increases as ALS progresses, and these cells produce IFN-γ, which affects CD8^+^ T cells’ survival and the killing of motoneurons (Andjus et al., 2009; Bataveljic et al., 2014; Coque et al., 2019). According to Coque et al. (2019) interaction between motor neurons and CD8+ T cells rely on the association between self-antigens and MHC class I molecules, though these autoantigens are still under investigation, and recent data suggest a clonal expansion of CD8+ T cells against an unknown antigen in fALS cases bearing mutation in the SETX gene. Conversely, CD8+ T cells expressing HLA-DR were reported to be increased in the blood and CSF of patients with sALS. Thus, it has been proposed that an ALS-associated antigen may initiate T-cell mediated autoimmune processes (Rodrigues Lima-Junior et al., 2021). In this context, Ramachandran et al. (2023) investigated reactivity of CD8+ T cells of ALS patients and their healthy counterparts against ten TDP43-derived peptides, However, the authors revealed that TFP43 is a weak autoantigen, and the investigations aiming to elucidate the autoantigens continues.

In a study published by Kaur et al. (2022), CD8+ T cells of ALS patients had increased CD28 and CCR7 expression in contrast to decreased IFN-γ receptor, which may be due to the constitutive IFN-γ expression. Promoting not only activation of the immune cells but also regulating the survival and function of NK and CD8+ T cells, absence or decrease in IFN-γ receptors on CD8+ T cells is an important finding which may lead to aforementioned increase in their lifespan and cytotoxic capacity to kill motoneurons.

CD8+ T cells were previously observed in the spinal cord of both ALS patients in addition to ALS SOD1G93A mouse models, and an *in vivo* study revealed that these cells infiltrate CNS at the symptomatic stage where selective ablation of CD8+ T cells in mice decreased spinal motoneuron loss (Ramachandran et al., 2023). Rolfes et al. (2021) reported an increase in CD8+ T cell frequencies expressing HLA-DR in peripheral blood and CSF in comparison with the control group. In this study, authors also underlined that T cell activation observed in the periphery coincided with increased intrathecal T-cell activation, suggesting that CD8+ T cells may contribute to blood/CSF-barrier dysfunction.

Although there is still not enough detailed data on the role and molecular mechanisms of CD8^+^ T cells in ALS, future studies should aim to elucidate these mechanisms and discover potentially new immunological mechanisms of CD8^+^ T cells in the etiopathogenesis of ALS, including their interactions with CD4^+^ T cells and cells of innate immunity. Understanding the role of CD8^+^ T cells in ALS could provide new therapeutic targets for treating this devastating disease.

### 3.3 T regulatory cells

T regulatory cells (T_regs_) are a subpopulation of T cells that play a crucial role in maintaining immune homeostasis and self-tolerance. They regulate the activity of other immune cells, such as T helper cells and cytotoxic T cells, by suppressing their activation and proliferation (Sakaguchi et al., 2010). Clinical studies on the potential neuroprotective function of T cells have revealed evidence of the protective function of CD4 + FOXP3+ regulatory T_regs_, with the quantity of Treg cells in the blood of ALS patients negatively correlated with the rate of disease progression (Henkel et al., 2013; Rolfes et al., 2021). Compared to healthy controls, T_regs_ increase at early, slowly progressing stages of ALS, and then their numbers as well as their immunosuppressive functions decline (Beers et al., 2017). According to Henkel and colleagues, T_reg_ numbers along with their FOXP3 protein expression were reduced in patients with rapidly progressing ALS (Henkel et al., 2006). In addition, authors have reported a reduction of the FoxP3 protein in post-mortem spinal cord of ALS patients with rapid progression, a GATA3 increase in patients with slower progression, suggesting that the decline in FOXP3 levels may be used as a marker for rapid progression. The reduction in T_reg_ cell number and function, along with the pro-inflammatory phenotype, contributes to the aberrant immune response and neuroinflammation observed in ALS. In a recent study, high proportion of activated T_regs_ in the peripheral blood of ALS patients were shown to be linked with better survival (Yazdani et al., 2022).

Even though these data underline the crucial role for T_reg_ cells in ALS pathogenesis, the therapeutic potential of T_reg_ cells in ALS highlights the need for further research in this area as clinical trials targeting these cells in ALS is still rare. In a phase 2 randomized trial, low-dose IL-2 has been reported to increase the percentage of T_regs_ (Camu et al., 2020). In another study, it was discovered that the infusion of expanded autologous T_regs_ revealed enhanced suppressive activity and slowed the disease progression (Thonhoff et al., 2018a,b). Further studies, on the other hand, are still needed in order to develop more effective cellular therapies including administration of T_regs_ in ALS.

### 3.4 The role of humoral immunity –antibodies in ALS

ALS patients are reported to have increased serum autoantibody levels compared to healthy controls (Provinciali et al., 1988). One study indicated increased levels of IgG and IgM, where the severity of the disease is correlated with higher antibody levels (May et al., 2014). Additionally, ALS patients with longer survival were reported to have IgM antibodies against modified, oxidized SOD1 protein compared to human control subjects (van Blitterswijk et al., 2011). Bataveljic et al. (2014) reviewed the humoral immunological markers, such as circulating immune complexes (CICs) and immunoglobulins. Saleh et al. (2009) assessed the humoral immune response during disease progression over 6 months, finding increased levels of CICs and IgG in the sera of ALS patients at the initial stage of the disease. After 6 months, in the same group of ALS patients, CICs were reduced to control levels, whereas IgG was increased compared to those in human control subjects. The presence of CICs is emphasized in various studies (Tavolato et al., 1975; Oldstone et al., 1976; Bartfeld et al., 1982).

Interestingly, the literature data differ concerning the levels of IgG in the sera of ALS patients. Some data suggest elevated IgG levels and normal IgM levels (Provinciali et al., 1988; Apostolski et al., 1991; Duarte et al., 1991; Saleh et al., 2009), while others do not detect any alterations in Ig levels (Bartfeld et al., 1982) or even detect a decrease in immunoglobulin levels (Zhang et al., 2005). Discrepant results could be due to different criteria for selecting ALS patients with different stages of the disease. Even in a study by Apostolski et al. (1991), it was shown that there is a significantly higher titer of IgG in the sera and CSF of patients with ALS (this study also showed a significantly increased mean value of the C4 component of the complement, as well as a significantly reduced value of THC – the total hemolytic titer complement). The results regarding the studies investigating the involvement of humoral immunity in ALS are rather inconsistent: in one study, no differences were found between the levels of IgG, IgM, IgA, in addition to the complement component C3 in ALS patients and controls (Bartfeld et al., 1982), whereas in another study, elevated mean serum IgG values were observed in a small group of ALS patients (Tavolato et al., 1975). Yet another study indicated increased levels of IgA and IgE in the sera of ALS subjects (Appel et al., 1986).

Numerous autoantibodies have been observed in ALS patients, such as antibodies against ganglioside GM1 (van Schaik et al., 1995) which localize near the nodes of Ranvier, and it is suggested that they are patient-specific for ALS patients with affected lower motor neurons (Pestronk et al., 1988). One study showed that the most frequently increased levels of anti-AGM1-gangliosides are in the sera of up to 1/3 of patients with ALS, whereas in the cerebrospinal fluid, the frequency of anti-GM1-gangliosides is higher compared to anti-AGM1-gangliosides and anti-sulfatides (Niebroj-Dobosz et al., 1999). This study revealed that ALS and other neurodegenerative diseases, as well as various forms of neuropathies such as non-sensory neuropathy, have a high concentration of anti-sulfatide antibodies. Additionally, in rare cases of ALS patients, elevated levels of antibodies against acetylcholine receptors were found (Mehanna et al., 2012). Autoantibodies targeting calcium channels (La Bella et al., 1997) and the Fas protein were also observed (Yi et al., 2000; Sengun and Appel, 2003), in addition to the antibodies against Low-Density Lipoprotein Receptor-Related Protein 4, which is essential for the development and function of the neuromuscular synapse (Tzartos et al., 2014). The Fas molecule, or CD95, is a membrane receptor that belongs to the TNF family and has a primary role in the transduction of programmed cell death (apoptosis) signals. It is highly represented in tissues and organs such as thymocytes, hepatocytes, kidneys, and the heart (Sengun and Appel, 2003).

Previously, it was reported that 25% of patients with sporadic ALS and 22% of patients with familial ALS had extremely high levels of anti-Fas antibodies. These high levels were observed not only in ALS but also in other neurodegenerative diseases. However, no relationship between anti-Fas antibody levels and disease duration or stage was found in ALS (Sengun and Appel, 2003). Another study has reported elevated levels of autoantibodies against neuronal cytoskeleton proteins (especially neurofilaments) in ALS patients, particularly during the later stages of the disease. Antibodies against light (NF-L) and heavy chain (NF-H) were found to be good surrogate markers of treatment response (Lu et al., 2011, 2012). However, anti-NF-L Abs were also elevated in other neuroinflammatory and neurodegenerative diseases such as multiple sclerosis (Fialová et al., 2009, 2012). The uniform distribution of antibodies to axonal cytoskeletal antigens in various biological fluids was shown to be a promising platform for discovering new biomarkers and developing immunomodulatory therapies not only for ALS but various neurological diseases (Fialová et al., 2009).

Immunoglobulins can induce pathophysiological processes at a nerve-muscle synapse by passive transfer, thus they can induce immune-mediated mechanisms in ALS pathogenesis (Bataveljić et al., 2011). When rodents were treated with IgG from ALS patients, the treatment led to an increase in intracellular calcium concentration, degenerative structural changes, and the induction of plasticity in nerve-muscular synapses (Uchitel et al., 1988; Engelhardt et al., 1995; Fratantoni et al., 2000; Pullen and Humphreys, 2000; Pullen et al., 2004; Pagani et al., 2006). The ALS IgGs have proven to suppress KCl-induced Ca^2+^ transients via selectively action on P/Q-type calcium channels *in vitro* (Andjus et al., 1996) in addition to exerting indirect effects (via phospholipase C) on N-type calcium channels (Pagani et al., 2006). Moreover, ALS IgGs can promote vesicle mobility and alter cytosolic Ca2+ homeostasis in cultured rat astrocytes (Stenovec et al., 2011; Milošević et al., 2013), induce glutamate release from primary rat hippocampal neurons (Andjus et al., 1997) and enhance oxidative stress while inducing antioxidant responses in a rat microglial cell line (Milošević et al., 2017).

Intravenous immunoglobulin G (IVIg) has been investigated as a potential treatment for ALS to its ability to modulate the immune response and its potential for protecting motor neurons from degeneration. Several clinical studies have been conducted to evaluate the safety and efficacy of IVIg in ALS patients. Both Meucci and coworkers and Dalakas and coauthors found that IVIg treatment had no therapeutic role on the symptoms related with ALS and did not slow the disease progression (Dalakas et al., 1994; Meucci et al., 1996). However, some clinicians still use IVIg in certain cases of ALS, particularly in patients with evidence of autoimmune dysregulation (Burrell et al., 2011). Further studies are needed to clarify the potential role of IVIg in the treatment of ALS.

### 3.5 Cytokines

Cytokines are small proteins, also known as humoral factors, secreted by cells that interact with other cells. Cytokines have three modes of action - autocrine (acting on themselves), paracrine (acting on nearby cells), and endocrine (acting over long distances) (Zhang and An, 2007). There are studies indicating the increase of both pro-inflammatory and anti-inflammatory cytokines in sera of *in vivo* ALS models as well as in ALS patients (Mitchell et al., 2009). In this context, the involvement of the cytokines IL-1ß, IL-6, IL-12, IL-15, IL-17, and IL-33 in ALS will be discussed.

#### 3.5.1 Interleukin-1β and-18

Members of the IL-1 family, IL-1β, is synthesized in its inactive form and activated by caspase-1 in response to various “danger” signals which are recognized by cytosolic inflammasome complexes (Martinon et al., 2009). Inflammasomes are defined as multimeric protein complexes that orchestrate caspase-1-mediated inflammatory responses including maturation and secretion of inflammatory cytokines IL-1β and IL-18, and eventually inflammatory cell death, pyroptosis (de Zoete et al., 2014).

The presence of active caspase-1 in the spinal cord and cerebral spinal fluid of ALS patients and ALS models of mice have been recognized more than two decades ago, and *in vivo* studies have revealed that caspase-1 or IL-1β depletion or IL-1β inhibition resulted in prolonged survival without affecting disease onset (Iłzecka et al., 2001). This study also suggested the involvement of caspase-1 activation in ALS pathogenesis. Based on the results of preclinical studies (Meissner et al., 2010; de Zoete et al., 2014), Maier et al. investigated the safety and efficacy of IL-1β antagonist Anakinra (ANA) (Maier et al., 2015). Authors reported that ANA is well tolerated and can be considered safe in ALS patients. However, ANA administration failed to inhibit the disease progression in comparison with the control group as measured by the ALSFRSr, and in addition, 94% of the ANA group developed anti-ANA antibodies. On the other hand, Italiani and coauthors reported that IL-18 is the only cytokine from the IL-1 family that correlates with ALS and indicated that further investigations are required to define if upregulation of IL-18 in ALS is either a consequence, or one of the causes leading to the disease pathology (Italiani et al., 2014). Both IL-1β and IL-18 are the products of NLR Family Pyrin Domain Containing 3 (NLRP3) inflammasome complex activation. In fact, activation of NLRP3 inflammasome complex has been previously reported in both microglia obtained from mouse models of ALS as well as ALS patients’ brain tissue samples, and TDP43 has been shown to be activate microglial NLRP3 (Johann et al., 2015; Zhao et al., 2015; Kadhim et al., 2016). In a more recent study performed with monocyte-derived microglia-like cells obtained from ALS patients, the cells were reported to have abnormally aggregated TDP43 in the cytoplasm, which is accompanied by an increase in IL-18 levels along with IL-8, TNF-α and TGF-β (Quek et al., 2022). However, when considering the involvement of NLRP3 inflammasome activation in ALS (Deora et al., 2020), more studies focusing on IL-18 may provide better insights if blocking inflammasomes may be a beneficial strategy in ALS treatment.

#### 3.5.2 Interleukin-6

IL-6 is one of the first cytokines which is found to be elevated in several neurodegenerative diseases including ALS (Sekizawa et al., 1998). Ehrhart et al. reported significant elevation of IL-6 levels in the blood samples in ALS patients; moreover, normalization of IL-6 along with IL-5 at the follow up was found to be associated with decreased IL-2 and increased IL-8 levels (Ehrhart et al., 2015), indicating that different humoral factors are involved in different inflammatory responses during the disease course. Similarly, Lu and coauthors reported an IL-6 increase in certain subgroups of ALS patients including male patients in contrast to females, patients with spinal onset, patients with slow progression, and patients receiving riluzole treatment (Lu et al., 2016). Interestingly, a correlation between IL-6 and the ALSFRS-R was reported in another study (Quek et al., 2022). On the other hand, a more recent study revealed that serum IL-6 levels are negatively correlated with ALSFRS-R in patients carrying IL6R358Ala variant (Wosiski-Kuhn et al., 2021).

Targeting IL-6 signaling has proposed as a therapeutic strategy in ALS: in a clinical trial, IL-6 receptor blocker tocilizumab (Actemra) was reported to decrease C-reactive protein (CRP) levels in plasma and CSF, in addition to being well tolerated and safe in ALS patients (Milligan et al., 2021). However, further studies are needed to conclude its benefits on the disease treatment.

#### 3.5.3 Interleukin-12 and-15

IL-12 and IL-15 are pro-inflammatory cytokines that are increased in the serum and cerebrospinal fluid of ALS patients. However, it has not been demonstrated that these interleukins have a mutual dependence on the duration of the disease (Rentzos et al., 2010). IL-12 is a heterodimer synthesized by monocytes, macrophages, dendritic cells, and microglia in response to various immunological and infectious signals (Ma and Trinchieri, 2001). Its role is to stimulate the proliferation and differentiation of T cells and the production of pro-inflammatory cytokines such as IFN-γ and TNF-α (Li et al., 2003). Monocytes produce IL-15 similarly to IL-12, as do macrophages, dendritic cells, and glial cells (Grabstein et al., 1994; Doherty et al., 1996; Lee et al., 1996; Jonuleit et al., 1997). IL-15 promotes the proliferation of T cells, induces NK cells, cytotoxic cells, as well as cells of humoral immunity by inducing the maturation of B cells and the secretion of antibodies (Carson et al., 1994). These data suggest that IL-12 and IL-15, which act through the Th1 response, may be associated, and involved in the pathophysiological mechanisms of ALS (Rentzos et al., 2010).

#### 3.5.4 Interleukin-17

According to literature, IL-17A regulates the responses of central nervous system resident cells, boosts the neuroinflammatory response, and contributes to the pathogenesis of neurodegenerative diseases. However, there is still debate and ambiguity surrounding the function of TH17/IL-17A in neurodegenerative diseases (Fu et al., 2022). IL-17 is produced by the Th17 cells and is implicated in various autoimmune and inflammatory diseases, and studies have shown that IL-17 may play a role in the pathogenesis of ALS. Along with IL-23, IL-17 levels were significantly increased in the serum and CSF samples of ALS patients, however the levels of the cytokines did correlate with the disease duration, disability scale or the subtype of the disease (Rentzos et al., 2010). Chen and coworkers indicated a significant association between ALS and IL17 levels (Carson et al., 1994). Fiala and coauthors reported IL17A^+^ cytotoxic T cells and mast cells in the spinal cords of ALS patients (Fiala et al., 2010). Regarding *in vivo* ALS models, IL-17A levels were found to be gradually increasing with age in SOD1G93A mice (Noh et al., 2014). More recently, Jin and coauthors reported the potential therapeutic benefits of anti-IL-17 treatment to alleviate neurodegeneration observed in ALS (Jin et al., 2021). Even though these findings suggest that IL-17 may be a potential therapeutic target for the treatment of ALS, currently there are no clinical data investigating the effect of targeting Th17 cells or IL17 in ALS.

#### 3.5.5 Interleukin-33

IL-33 is a cytokine that functions as an alarmin, which is released from damaged tissue. This cytokine is one important cytokine responsible for the shift in T cell responses and stimulates innate type 2 immune cells to produce Th2 cytokines (Korhonen et al., 2019). IL-33 receptor, ST2, can exist in the membrane or soluble form. In a study evaluating the levels of IL-33 in addition to its soluble receptor revealed significantly reduced levels of IL-33 and elevated levels of sST2 in ALS patients (Lin et al., 2012). The reason for the reduced levels of IL-33 is that motor neurons in ALS die through programmed cell death of the apoptosis type, and IL-33 is degraded by the action of the executioners of apoptosis, i.e., by the action of caspases (Sathasivam et al., 2001). It is also known that the sST2 receptor can act as a positive or negative feedback regulator, suppressing or stimulating cytokine expression. Furthermore, one study suggested that the sST2 receptor acts as a degradation receptor by inhibiting IL-33-mediated signaling (Miller, 2011). However, the regulation of sST2 receptor levels has not been fully clarified (Lin et al., 2012).

*In vitro* studies have suggested that instead of directly acting on neurons and astrocytes, IL-33 regulates responses of peripheral T cells (Korhonen et al., 2019). *In vivo*, long-term recombinant IL-33 treatment delayed the disease onset on female mice in a transgenic mouse model of ALS expressing SOD1-G93A while male mice remained unresponsive. These findings indicate that strategies targeting to modulate the peripheral immune system may have therapeutic potential in ALS, and the effect of the gender should be considered when designing therapeutic strategies. It should be noted that further research is required to determine the ideal method for delivering IL-33 to the CNS in order to reduce inflammation without causing adverse effects in other organs since IL-33 is a potent cytokine (Sun et al., 2021).

## 4 Conclusion remarks and future perspectives

This review highlights the complex interplay between neuroimmunology and neuroinflammation in the pathogenesis of ALS, which is a devastating disease that currently has no effective cure. Several points were highlighted regarding innate immunity, including the limited information available on the monocyte’s role in ALS, the diversity of microglia as either neurotoxic or neuroprotective, the complex role of the complement system in ALS, and the contribution of NK cells, NKT cells, mast cells, neutrophils, and astrocytes. From the perspective of adaptive immunity, CD4^+^T cells were identified as early disease markers possessing a neuroprotective identity, while CD8^+^T cells were late disease markers with cytotoxic properties. The role of humoral immunity, specifically IgG and IgM, needs further clarification, and the contribution of autoantibodies to ALS neurodegeneration requires better understanding.

Our review suggests that a more comprehensive understanding of the neuroimmunology and neuroinflammatory aspects of ALS will pave the way for the development of new therapeutic strategies for this devastating disease. Moving forward, future research should focus on exploring the potential of NKT cell-targeted therapies, identifying potential biomarkers in ALS, clarifying the role of IVIg in treatment, and further investigating the impact of alterations in the gut microbiome on ALS pathogenesis. Additionally, expanding research to include more context on adaptive immunity, CD8^+^T cells, and autoantibodies may lead to a more complete understanding of the immune response in ALS and instate the development of new therapeutic approaches. Overall, continued research into neuroimmunology and neuroinflammation in ALS has the potential to improve patient outcomes and provide hope for those suffering from this devastating disease.

## Author contributions

SM: Conceptualization, Data curation, Investigation, Visualization, Writing – original draft. BA: Conceptualization, Data curation, Investigation, Visualization, Writing – original draft. CP: Conceptualization, Data curation, Investigation, Visualization, Writing – original draft. HS: Data curation, Investigation, Writing – original draft. PA: Conceptualization, Funding acquisition, Project administration, Resources, Supervision, Writing – review & editing. GYD: Conceptualization, Funding acquisition, Project administration, Resources, Supervision, Writing – review & editing.
